# Conducting research with Indigenous Peoples in Canada: ethical and policy considerations

**DOI:** 10.3389/fpsyg.2023.1214121

**Published:** 2024-01-10

**Authors:** Dominique Morisano, Margaret Robinson, Brian Rush, Renee Linklater

**Affiliations:** ^1^Dalla Lana School of Public Health, University of Toronto, Toronto, ON, Canada; ^2^Department of Psychology, University of Ottawa, Ottawa, ON, Canada; ^3^Institute for Mental Health Policy Research, Centre for Addiction and Mental Health, Toronto, ON, Canada; ^4^Departments of English and Sociology & Social Anthropology, Dalhousie University, Halifax, NS, Canada; ^5^Shkaabe Makwa, Centre for Addiction and Mental Health, Toronto, ON, Canada

**Keywords:** Indigenous, policy, ethics, research, methodologies, First Nations, principles, guidelines

## Abstract

The international context of Indigenous mental health and wellbeing has been shaped by a number of key works recognizing Indigenous rights. Despite international recognitions, the mental health and wellness of Indigenous Peoples continues to be negatively affected by policies that ignore Indigenous rights, that frame colonization as historical rather than ongoing, or that minimize the impact of assimilation. Research institutions have a responsibility to conduct ethical research; yet institutional guidelines, principles, and policies often serve Indigenous Peoples poorly by enveloping them into Western knowledge production. To counter epistemological domination, Indigenous Peoples assert their research sovereignty, which for the purposes of this paper we define as autonomous control over research conducted on Indigenous territory or involving Indigenous Peoples. Indigenous sovereignty might also be applied to research impacting the landscape and the web of animal and spiritual lives evoked in a phrase such as “all my relations.” This narrative review of material developed in the Canadian context examines the alignment with similar work in the international context to offer suggestions and a practice-based implementation tool to support Indigenous sovereignty in research related to wellness, mental health, and substance use. The compilation of key guidelines and principles in this article is only a start; addressing deeper issues requires a research paradigm shift.

## 1 Introduction

The International context of Indigenous mental health and wellbeing has been shaped by a number of key policies and guidelines recognizing Indigenous rights. Most significant among these is the *United Nations Declaration on the Rights of Indigenous Peoples* (UNDRIP, 2007), whose 46 Articles outline “minimum standards for the survival, dignity and wellbeing of the Indigenous Peoples of the world” (p. 28, Article 43). A motion to adopt the Declaration was passed by the UN General Assembly over the objections of four states (Australia, Canada, New Zealand, and the United States), each of which have reversed their objections. The UNDRIP recognizes that Indigenous Peoples are distinct and come from self-determining nations who require free, prior, and informed consent when interacting with other countries, nations, and foreign governments. UN Declarations are generally not legally binding; however, they represent the dynamic development of international legal norms and reflect the commitment of nations to move in certain directions, abiding by certain principles. Canada was the last to sign on to adopt the UNDRIP into Canadian law in June 2021, this process being led by the Province of British Columbia in 2019.

International declarations such as the United Nations Declaration on the Rights of Indigenous Peoples (UNDRIP) (2007) supersede and encompass national or regional guidelines designed to govern research, and are frequently invoked in arguments at the national and regional levels. Despite such International recognitions, the wellbeing and mental health of Indigenous Peoples continue to be negatively affected by policies and guidelines that ignore Indigenous rights, that frame colonization as historical rather than ongoing, and that minimize the impact of assimilation (George et al., 2019). Nor is the tokenization and deprioritizing of Indigenous health limited to policymakers. A systematic review of academic literature from 2000 to 2015 found that of 210 articles about circumpolar environment-related Indigenous health, only 39 (19%) reported engaging with Indigenous Peoples (Jones et al., 2018).

Research institutions of all kinds have a responsibility to conduct ethical research, yet institutional engagement of principles and guidelines often has served Indigenous Peoples poorly, enveloping them inside Western knowledge production. As stated by Cree scholar Willie Ermine and his colleagues, “the way research is talked about assumes that all research is properly undertaken from the perspective and under the auspices of Western centers of authority” (Ermine et al., 2004, pp. 28–29). To counter this epistemological domination, Indigenous Peoples assert their research sovereignty, which for the purposes of this paper we define as autonomous control over research conducted on Indigenous territory or involving Indigenous Peoples. Given the relational worldview of many Indigenous nations, Indigenous sovereignty might also be applied to research impacting the landscape and the web of animal and spiritual lives evoked in a phrase such as “all my relations.” This narrative review essay draws from guides and documents developed in the Canadian context to offer international policy and practice lessons to support Indigenous sovereignty in mental health and substance use health-related research.

The compilation of key principles and guidelines^1^ in this article is only a start; addressing deeper epistemological issues requires a research paradigm shift. Traditional goals of extensive and frequent publishing, the reality of short deadlines for grant applications, and the urgent demand for results do not always mesh well with the requirements for conducting research ethically (Castleden et al., 2015). As discussed in Willie Ermine et al.’s (2004) work for the Indigenous Peoples’ Health Research Centre (IPHRC) and in Smith’s (1999) seminal book *Decolonizing Methodologies*, the term “research” represents a negative and harmful enterprise to many Indigenous Peoples. As Smith (1999) noted, it is inextricably linked to European imperialism and colonialism, making it “one of the dirtiest words in the Indigenous world’s vocabulary” (p. 1). Indeed, settler directed research has exhibited a lack of knowledge about Indigenous histories, methodologies, and worldviews, subsequently aligning poorly with the goals and values of Indigenous Peoples (Fournier et al., 2023). As a result, research has produced little or no benefit for Indigenous communities and their members (Hyett et al., 2018), and in some cases has reinforced deficit-based views of Indigenous Peoples (Mashford-Pringle and Pavagadhi, 2020). Mosby (2013) has drawn attention to research conducted without consent on Indigenous children held in Residential Schools; and a 2021 class action lawsuit filed against two Canadian medical researchers (Moore, 2021) charged that they conducted magnetic resonance imaging of the livers of Indigenous Peoples (including a First Nation Chief) without their knowledge or consent.

## 2 Methods

While working for the Centre for Addiction and Mental Health (CAMH)^2^ in Toronto, Canada, three of the authors for the present paper conducted a narrative review of over 120 scholarly articles, policy-oriented documents, books, and online resources that offered guidelines for Canadian settlers conducting mental health research with Indigenous Peoples. This work was summarized in a CAMH internal report (Morisano et al., 2018). To facilitate feedback on that report, a large advisory committee of First Nations and Métis scholars and clinicians was recruited from CAMH and from other non-profit institutions in Toronto (see Acknowledgments section of Morisano et al., 2018), and the current article draws upon their contributions.

For both the original report (Morisano et al., 2018) and this paper, considerable efforts were made to seek and prioritize Indigenous voices and authorship in the collected works. Sources were identified via mixed methods, including works known to the authors themselves, queries with online search engines (Google, DuckDuckGo), academic databases (Scopus, Google Scholar), bibliographic review of found documents, and via original books and non-published works recommended by the advisory committee and external scholars. Source criteria included that each document was (a) in active use in health research circles during the time period of our review (2012–2023, for this paper); (b) developed by or with considerable engagement from Indigenous (First Nations, Métis, Inuit) Peoples; and (c) relevant to health research work being conducted with Indigenous Peoples in the fields of substance use and wellness, and/or mental health.^3^ The majority of sources for this paper were published^4^ by Indigenous researchers for the purpose of changing dialogue and practice when conducting health research with Indigenous Peoples.

The original report (Morisano et al., 2018) aimed to provide CAMH researchers with a comprehensive overview of relevant research guidelines and principles and provide a synthesis to guide their work. The report identified four foundational documents and 14 principles to guide mental health research, and examined nine Indigenous-led research projects for how they embodied those principles. It also provided an overview of three Indigenous-developed ethical review systems (i.e., Institutional Review Boards) and six methods commonly used in Indigenous health research.

The current article differs from the report from which it evolved (Morisano et al., 2018) in several important ways. Not only has it expanded to address specific policy considerations, but it has also been heavily updated, significantly condensed, and synthesized into themes for an international research audience. Although the focus is on works most relevant to health research, the principles may apply across a range of research disciplines. Thematic analysis aided the authors in distilling key principles and themes from the original sources.^5^ The present paper focuses on four themes common to the Canadian literature and policy context, and their implications for international work: (1) respect for Indigenous governance and culture; (2) meaningful engagement and collaboration; (3) utility of research; and (4) collective ownership. We also provide a supplementary tool: a list of reflective questions to support planning and implementation of ethical research with Indigenous Peoples.

## 3 Themes in the Canadian literature on mental health and wellness research with Indigenous Peoples

Our analysis of Canadian research guidelines identified four common themes, the first of which is respect for Indigenous governance and culture.

### 3.1 Respect for Indigenous governance and culture

The Canadian Institutes of Health Research (CIHR) spend approximately $1 billion annually to fund health-related research, and that research is guided by policy published jointly with two other national funding bodies, the Social Sciences and Humanities Research Council (SSHRC) and the Natural Sciences and Engineering Research Council (NSERC) (collectively referred to as the Tri-Council). Their document, *Ethical Conduct for Research Involving Humans,* 2nd edition (Tri-Council Policy Statement or TCPS-2; Tri-Council, 2022), contains a section titled “Research Involving the First Nations, Inuit and Métis Peoples of Canada.”^6^ That chapter—colloquially referred to as ‘chapter nine,’ offers guidelines akin to Australia’s *Ethical Conduct in Research with Aboriginal and Torres Strait Islander Peoples and Communities: Guidelines for Researchers and Stakeholders* (National Health and Medical Research Council, 2018a).^7^

The authors of the TCPS-2 note that working with Indigenous communities or organizations may entail navigating “complex authority structures” (Tri-Council, 2022; article 9.5). In some communities, authority to permit and monitor research lies with individuals designated via custom rather than appointment or election. The Tri-Council suggests researchers engage community processes and Elders to determine how to secure approval for research activity in a community (article 9.15). The Tri-Council recommends securing approvals from both customary authorities and formal leaders. Engaging authority figures often involves presenting tobacco,^8^ sage, or other small gifts; offering honoraria, a donation in their name, and/or name recognition; covering travel expenses; and/or using an interpreter so authorities can speak in their Indigenous language (Noojmowin Teg Health Centre of Manitoulin Island, 2003; Wilson, 2008; First Peoples Cultural Council, 2021; Kovach, 2021; Tri-Council, 2022, article 9.15). The First Nations Information Governance Centre (FNIGC, 2020) released a *First Nations Data Governance Strategy* with a mission to assert “data sovereignty and support the development of information governance and management at the community level through regional and national partnerships” and to “adhere to free, prior and informed consent, respect nation-to-nation relationships, and recognize the distinct customs of nations” (p. 2).

The authors of the *Principles of Ethical Métis Research* (Métis Centre of National Aboriginal Health Organization, 2011) discuss the importance of respect for individual and collective perspectives in research processes (and the straddling of these perspectives depending on the research proposed). They suggest that researchers seek out and follow community practices and protocols. The Inuit Tapiriit Kanatami (ITK) and Nunavut Research Institute (NRI) guide (2006) recommended researchers “assign the same value, credibility, and respect to local expertise (from recommended Elders, or others) as that assigned to peer-reviewed scientific findings” (p. 5). From an Inuit perspective, the authors pointed out the importance of not disturbing families “on particular days of the week, times of day, or in the wake of a local tragedy” (ITK & NRI, p. 8) and reminded researchers that for most communities, “research is fairly secondary as local life and activities continue” (ITK & NRI, p. 8).

In situations where work with Indigenous Peoples is planned but no Indigenous governance structures exist (e.g., in an urban community), community agencies or institutions can be consulted. As an example of a local consultation process, Toronto Aboriginal Support Services Council (TASSC), a group of members from service agencies in the Toronto area, oversaw a large community-based research initiative called the Toronto Aboriginal Research Project (TARP) from beginning to end. TARP was initiated to provide an “extensive picture of the current situation, successes, aspirations, and challenges facing Aboriginal People in the Greater Toronto Area” (McCaskill et al., 2011, p. 17). As of the time of publication of this article, the collection of data by TASSC for TARP 2.0^9^ was in process. Additionally, the Noojimawin Health Authority (n.d.) (NHA) was a Toronto-based Aboriginal Health Planning Authority that aimed to improve health conditions for rural and urban Aboriginal People. Before closure, they published an *Ethical Research Policy for Urban and Rural Aboriginal Health* (n.d.), offering principles and procedures to guide themselves and their partners in “respectful research practices in urban and rural areas in the province of Ontario with respect to Aboriginal health” (p. 3). Their document reviewed multiple domains: protecting Indigenous Knowledge, respecting Indigenous Knowledge and experience, the idea of research as partnership, the use of research agreements and the creation of memoranda of understanding, consent processes, collecting and sharing data, ensuring the community benefits and the sharing, dissemination and publication of research results, and implementation of findings in the communities. Furthermore, The Ontario Federation of Indigenous Friendship Centres (OFIFC), which comprises Ontario’s largest urban service network, released the 2nd edition of their *Utility Self-Voicing Access Inter-relationality (USAI) Research Framework* in 2016, to guide all Indigenous research projects involving the Ontario Federation of Indian Friendship Centres (2016). In 2018, the Urban Indigenous Health Research Gathering was hosted in Manitoba by the Ongomiizwin-Indigenous Institute of Health and Healing at the University of Manitoba to “engage urban Indigenous People with a variety of perspectives and experiences to learn about engaging urban Indigenous communities in health research” (p. 4) and report on the findings (Morton, 2019).

Indigenous Peoples in urban environments generally exhibit interhousehold variation in socioeconomic status and the extent to which they engage in traditional practices—the definition of “traditional” itself is also dependent on one’s context and lens (Lindstone, 2014). Some individuals may have stronger affiliations to their First Nation, Inuit, or Métis community or to a national or regional representative organization; others might be disconnected from those organizations. Similarly, a number of Indigenous Peoples do not make use of or consider themselves to be represented by Friendship Centres^10^ or other Indigenous service providers. Furthermore, there are issues even in the identification of issues faced by many First Nations, Inuit, and Métis persons in Canada (see Smylie and Firestone, 2015), given how data are collected for major health and social data sources. The authors noted a need for revision of core data health services in Canada, “in partnership with Indigenous Peoples and their representative and governing organizations” (p. 67).

#### 3.1.1 Respect for diversity

A set of *Guidelines for Ethical Aboriginal Research* localized to Manitoulin Island, Ontario, called the GEAR (Noojmowin Teg Health Centre of Manitoulin Island, 2003) made note of the need for research projects to “respect the diversity between and within communities” (p. 7). These concerns were echoed in the Métis research community (Métis Centre of National Aboriginal Health Organization, 2011). It is important not to make assumptions about language, worldviews, beliefs, politics, geographic orientation, cultural values, history, religion, or a variety of other factors when approaching individuals, organizations, or communities in the name of research. Many times, individuals engaged in research with Indigenous Peoples fail to consider the complex intersections of identity that are present, such as age, gender, sexual orientation, and socioeconomic status. An intersectional lens is required to understand the diverse experiences of Indigenous Peoples. There is diversity in diversity, and the multiplicity of documents reviewed in this paper is a testament to this fact. Even source documents focusing on specifically First Nations, Inuit, or Métis Peoples as distinct groups reminds us of the heterogeneity of Indigenous Peoples, and reinforces the idea that we are not working with a single homogeneous North American Indigenous category; but rather groups of people who stretch across a vast swath of land, with regional variances, and whose interactions with state, provincial, and federal governments have differed.

#### 3.1.2 The research team

Many current ethical guidelines recommend the research team of any project include members of the population of interest in meaningful roles (e.g., Ermine et al., 2004; Tri-Council, 2022). This should be negotiated at the outset of proposed research, as appropriate, and depending on the interest of the communities involved.

#### 3.1.3 Research design

The research design process in an Indigenous research project may differ from Western research processes taught in most Canadian educational institutions. Several guideline documents strongly encourage researchers to involve and/or partner with Indigenous community members in project design and delivery (Ermine et al., 2004; Inuit Tapiriit Kanatami and Nunavut Research Institute, 2006; First Nations Centre, 2007; Métis Centre of National Aboriginal Health Organization, 2011; The First Nations Information Governance Centre, 2014). Individuals or communities may not be interested in being involved at this level, but in a collaborative context, the option for such involvement should be available.

A key in this process is that researchers approach communities with research questions that are open to change, refinement, or correction (Nakamura, 2010). Ray and Cormier (2012) argued, for example, that the practice of designing an interview guide based on a pre-constructed hypothesis or research question conflicts with the Anishinaabe view in which knowledge is controlled by the knowledge holder rather than the knowledge seeker. Within such an approach, it would be the interview participants who determine what is important to share, based on their sense of the researcher’s level of understanding and readiness to carry the teaching. Therefore, taking the time to develop trust and mutual understanding with knowledge holders, before requesting knowledge, will result in better data.

Bartlett et al. (2007) suggest that Indigenous knowledge begins with a narrative that is transformed and personalized, whereas Western knowledge begins with data that are transformed into abstract knowledge. Indigenous Peoples might differ in their beliefs about what constitutes data and might include dreams, visions, intuition, and cellular or blood memory as sources of knowledge (Cordero, 1995; Castellano, 2000; Cardinal, 2001; Steinhauer, 2002; Loppie, 2007; Braun et al., 2013; Kovach, 2021). Research design should therefore incorporate relevant Indigenous views on information gathering and the nature of knowledge.

### 3.2 Meaningful collaboration

Despite variation in approaches to research among Indigenous nations, the importance of meaningful collaboration emerges as a highly significant theme. Attending to process--how collaboration is done and how knowledge is generated--comes across as a value embedded in multiple documents. For example, the Ojibwe phrase *Kinoo’amaadawaad Megwaa Doodamawaad,* which means “they are learning with each other while they are doing” (Cormier, 2016, p. 229), encapsulates the importance attributed to participatory approaches to research creation and could be taken as a description of meaningful collaboration itself. Below we detail six starting points for such collaboration.

#### 3.2.1 Use of community guidelines

The use of relevant community research guidelines is framed as necessary. Such guidelines require researchers to interact with the people they are seeking knowledge about, take training in cultural competence, learn new protocols and traditions, and create a culturally relevant research process (Indigenous Peoples’ Council on Biocolonialism, 2000; Ermine et al., 2004; First Nations Centre, 2005, 2007; Métis Centre of National Aboriginal Health Organization, 2011; The First Nations Information Governance Centre, 2014; Kovach, 2021). The onus is on the researcher to familiarize themselves with the growing body of literature on the topic. Many research ethics documents have been developed at the local, regional, and national levels by First Nations, Inuit, and Métis Peoples. Several of these guidelines have been published online, and additional guidelines are housed locally; some may be preserved through oral tradition only. In any case, local processes must be sought out and respected when conducting research in any community.

#### 3.2.2 Collaboration

Research conducted in Indigenous contexts must be collaborative and seen as building a meaningful partnership with communities. The TCPS-2 emphasized community engagement prior to embarking on specific research projects with those communities (Tri-Council, 2022). In many documents written by Indigenous researchers (Ermine et al., 2004; First Nations Centre, 2005, 2007; Inuit Tapiriit Kanatami and Nunavut Research Institute, 2006; Bull, 2010, 2016; Métis Centre of National Aboriginal Health Organization, 2011), relationship building is framed as something that should occur prior to design development and institutional approval. Investigators must see their projects as being both community-based and “community-paced” (First Nations Centre, 2007). According to the National Inuit Strategy on Research (NISR) from the Inuit Tapiriit Kanatami (ITK) (2018), “Inuit and researchers have reaped the benefits of research relationships premised on respect for Inuit self-determination and are seeking coherent and consistent research relationships across Inuit Nunangat” (p. 3). Communities should be consulted for their involvement, participation, and consultation (First Nations Centre, 2005, 2007; Inuit Tapiriit Kanatami and Nunavut Research Institute, 2006; The First Nations Information Governance Centre, 2014; Ontario Federation of Indian Friendship Centres, 2016; Inuit Tapiriit Kanatami, 2018). Examples of this kind of work have come from the Aboriginal Healing Foundation (Aboriginal Healing Foundation, 2010). The AHF was established in 1998 to fund “community-based Aboriginal directed healing initiatives which address the legacy of physical and sexual abuse suffered in Canada’s Indian Residential School System, including inter-generational impacts” (Aboriginal Healing Foundation, 2017). While the AHF ceased operations in 2014, its approach to research collaboration “require[d] a participatory process in which Aboriginal People determine how the AHF can most effectively respond to their healing needs” (Aboriginal Healing Foundation, 2017).

The TCPS-2 notes that “although researchers shall offer the option of engagement, a community may choose to engage nominally or not at all, despite being willing to allow the research to proceed” (Tri-Council, 2022, article 9.10). This is something that researchers must understand and of which they must be considerate. In cases where communities disengage because they lack the capacity (financial or otherwise) to participate fully, the TCPS-2 recommends that researchers spend additional resources supporting these communities in capacity building (see article 9.14). If there is no possibility for gaining community consent, collaboration, or a research agreement, but the researchers are still allowed to proceed, individual consent guidelines still apply.

#### 3.2.3 Consent, inclusivity, and approvals

Many ethical issues stem from the category of “research consent and approvals” in Indigenous contexts. Some of these relate to the appropriateness of gathering oral versus written consent. In general, any kind of information being collected from an individual must be explained in a language (with translation, as needed or desired) and manner that ensures fully informed consent (First Nations Centre, 2005; Inuit Tapiriit Kanatami and Nunavut Research Institute, 2006; Métis Centre of National Aboriginal Health Organization, 2011; Tri-Council, 2022).

Other issues that relate to consent concern the idea that a signed consent form does not represent a completed process (Ermine et al., 2004; Tri-Council, 2022). Many of the documents that discuss the ethics of conducting research with Indigenous Peoples frame the consent process in a circular and continuous manner that extends beyond a one-time signature.

Piquemal (2001, as cited in Ermine et al., 2004) makes four ethical recommendations for an informed-consent process: to negotiate responsibilities at the outset, to obtain consent from both collective and individual authorities, to confirm consent throughout the process to ensure that it is ongoing, and to provide the community with data at the end of any project. The TCPS-2 includes the idea that:

“Indigenous codes of research practice go beyond the scope of ethical protections for individual participants. They extend to the interconnection between humans and the natural world, and include obligations to maintain, and pass on to future generations, knowledge received from ancestors as well as innovations devised in the present generation.” (Tri-Council, 2022, chapter 9, “Introduction”).

In the Inuit context, Inuit Tapiriit Kanatami and Nunavut Research Institute (2006) guide suggests that any study be discussed first with local authorities (e.g., Hamlet Council, local Hunters and Trappers Organization) regarding requirements for consent, confidentiality, and adherence to institutional ethics protocols. Guidelines developed in response to the OCAP®^11^ principles of *ownership, control, access, and possession* that relate to consent have included the following: “Researchers should provide ongoing explanations of all aspects of the research project, including its purpose, sponsorship, anticipated benefits and risks, methods, community and individual involvement, and reporting plans” (First Nations Centre, 2005, p. 12). Secondary use of data that can be identified as coming from a particular Indigenous community or Peoples are still subject to requirements related to informed consent and/or community engagement, depending on the circumstance (see TCPS-2 article 9.20; Tri-Council, 2022). Ermine et al. (2004) noted that researchers should obtain approval to do research in Indigenous communities from the appropriate national Tribal authorities. In the case of requesting consent from urban, non-status, or displaced Indigenous Peoples without a governance structure, researchers may navigate the consent process with local community agencies or Indigenous urban organizations to assure an appropriate process is followed. Research involving historical, genealogical, or secondary data analyses on publicly available information that does not involve new data collection may not require Research Ethics Board (REB) review or community engagement, but it is suggested that “culturally informed advice” be sought before the use of such data to determine potential harms and other considerations (see TCPS-2 articles 9.15 and 9.21; Tri-Council, 2022).

Article 9.6 of the TCPS-2 discusses the importance of recognizing “diverse interests within communities,” including the inclusion of groups or individuals in research who may have been excluded from previous research opportunities due to vulnerability or marginalization within a community (Tri-Council, 2022). The Métis Centre of National Aboriginal Health Organization (2011) also noted the importance of “safe and inclusive environments” in research, and specified that age (youth and Elders), gender, sexual identity, multiple concepts of “Aboriginality,” and a “balance of individual and collective influence” be considered in research settings with Métis People (p. 2). Decisions for research exclusion or inclusion of a group or community must be made with care. In this same regard, when “critical inquiry” is made regarding First Nations, Inuit, and Métis governments, institutions, or authority structures, the Tri-Council (2022) suggests researchers consult regional or national organizations that are culturally relevant to Indigenous Peoples for guidance (see TCPS-2 article 9.7).

There are controversies related to the concept of “informed consent” that should be addressed. According to Ermine et al. (2004), “For Indigenous Peoples, the Western paradigm of individualism that recognizes the right of the individual to give knowledge through ‘informed consent’ is contradictory to the concept of collective ownership understood by Indigenous Peoples” (p. 30). Some guidelines suggest obtaining group or community consent before moving to obtain individual consent for research participation (see Ermine et al., 2004; Inuit Tapiriit Kanatami and Nunavut Research Institute, 2006; First Nations Centre, 2007). Ermine and colleagues propose that the concept of free and informed individual consent is problematic in Indigenous contexts as it “rests on the condition of Western sensibilities of the legal individual and individuality” (2004, p. 31).

#### 3.2.4 Community advisory boards

Community advisory boards for research are often composed of Elders or other traditional knowledge keepers familiar with Indigenous ethics and protocols, interested community members, and other volunteers (Ermine et al., 2004; Inuit Tapiriit Kanatami and Nunavut Research Institute, 2006). Indigenous Peoples may be sought as co-principal investigators, co-investigators, consultants, or collaborators on research projects (First Nations Centre, 2007; The First Nations Information Governance Centre, 2014; ITK, 2018). To date, many boards developed for Indigenous research are largely informal structures created by the researchers involved. Indigenous organizations and communities may create their own research advisory boards to ensure protocols are followed, and some communities have done this already (e.g., the Native Council of Prince Edward Island and NunatuKavut Community Council; see “Our Health Counts: Urban Aboriginal Health Database project,” below; Smylie et al., 2011). The Indigenous Peoples’ Council on Biocolonialism’s (Indigenous Peoples’ Council on Biocolonialism, 2000) *Indigenous Research Protection Act* suggests it is “in the best interest of the Tribal community to establish a research review mechanism to prevent the continued abuses, to protect the People’s traditional knowledge and properties, and thereby to ensure our rights to continue to practice traditional lifeways and long-term survival thereof” (Indigenous Peoples’ Council on Biocolonialism, 2000, s. 1.5). The IPCB was established to help “Indigenous Peoples in the protection of their genetic resources, Indigenous knowledge, cultural and human rights from the negative effects of biotechnology” (Indigenous Peoples’ Council on Biocolonialism-b, n.d.). They also recommend that an administrative fee be set by the community or organization to charge researchers for proposal review (see *Indigenous Research Protection Act*, section 6.3). In the Métis context, “community involvement” is framed as coming in the form of “knowledge of local customs, input into the research design, utilizing community members in the research process... etc.” (Métis Centre of National Aboriginal Health Organization, 2011, p. 1). In general, it is key to re-envision the way in which “experts” are defined and valued in traditional Western academic and non-academic research contexts, and to imagine the term “expert” encompassing a broad range of individuals with an expansive and diverse range of skills, knowledge, and ideas.

#### 3.2.5 Agreements or memoranda of understanding

In general, Indigenous research guidelines in Canada exhibit a move toward embracing “research agreements,” including “data sharing agreements.” The Tri-Council (2022) states, “Where a community has formally engaged with a researcher or research team through a designated representative, the terms and undertakings of both the researcher and the community should be set out in a research agreement before participants are recruited” (see TCPS-2, article 9.11). Ermine et al. (2004) connect research agreements with their concept of ethical space:

Formal research agreements are products of the ethical space where negotiation, dialogue, and discussions have taken place between cross-cultural entities. The aim of the negotiation process is to come to a clear understanding, which results in a formal agreement (preferably written) about research intentions, methods and potential results.… Issues like written documentation of consent from communities; status of ownership, control, access and possession of knowledge, data, information, and dissemination of findings through reports, and publication can be covered under these agreements (Ermine et al., 2004, p. 41).

There is a general sense in Indigenous research guidance documents that when it comes to research agreements, “there are no right answers, only options to explore and practical decisions to be made considering the nature of the information and the interests of the parties” (First Nations Centre, 2005, p. 32). The Inuit Tapiriit Kanatami and Nunavut Research Institute (2006) guide to working in Inuit communities suggests that any negotiated research relationship involves being honest, humble, informed, open, patient, and that researchers be willing to learn; educate locally; hire and purchase locally; maintain communication; respect local cultures, customs, and authority; try new things; and use or try to learn the local language. The Indigenous Peoples’ Council on Biocolonialism (2000) *Indigenous Research Protection Act* proposes that any good research agreement be based on mutual respect between “the researchers and the Tribe” (see section 5.1 h) and includes a section discussing guidelines for any created agreement (see section 8). The TCPS-2 notes that minimally, “the agreement should address the ethical protections that would apply to securing individual consent for a comparable project, and should specify any commitments regarding collective community participation and decision making, sharing of benefits and review, and updating of the agreement” (Tri-Council, 2022, article 9.11). Such agreements would “maximize the distribution of information while protecting sensitive information” (First Nations Centre, 2005, p. 25). An example cited by First Nations Centre (2005) included a discussion and template for negotiating research relationships prepared for Dene and Métis Peoples in the Northwest Territories in the early 1990s (Masazumi and Quirk, 1993). Research agreements can clarify the relationship between a community or organization and any research partners. The TCPS-2 makes multiple references to the incorporation of mutual expectations and obligations into a research agreement and suggests a research agreement may be one form of “evidence” for an REB to consider whether a researcher’s chosen plan of community engagement is appropriate (see Tri-Council, 2022, article 9.10). In discussions of informed consent, it states, “Where researchers and organizational communities or communities of interest collaborate in research (e.g., through a research agreement), prospective participants shall be informed about the extent of such collaboration (including how data will be shared) as part of the initial and ongoing consent process” (article 9.4). Where data-sharing agreements exist that allow community partners access to identifiable personal data, consent processes must reflect the disclosure (Tri-Council, 2022).

Under the now-retired Canadian Institutes of Health Research (2010) *CIHR Guidelines for Health Research Involving Aboriginal People*, the use of research agreements was emphasized for projects conducted with or about Indigenous groups. A template example is provided on its website (see Canadian Institutes of Health Research, 2010). The IPCB’s website also provided a template for use in creating academic contracts or research agreements (see Indigenous Peoples’ Council on Biocolonialism-a, n.d.).

For a variety of reasons, not all communities will be interested in signing a contract with researchers regarding impending projects. It is possible to keep research agreements brief and open to clarification, particularly in less formal arrangements (TCPS-2; Tri-Council, 2022). Furthermore, allowances can be made for semi-regularly revisiting such agreements to ensure that Indigenous research collaborators remain satisfied and fulfilled.

#### 3.2.6 Research ethics boards

In conjunction with institutional REBs, formal research ethics approval by local ethics boards may be required. For instance, in Ontario, the Six Nations Elected Council (2015) published a formal research ethics policy that applies to all research conducted on Six Nations of the Grand River Territory. The Six Nations Council Research Ethics Committee had already implemented a formal protocol and review process to be completed prior to any study’s initiation (see Six Nations Elected Council, 2009). As an example of impact, in 2018 McMaster University (Ontario) published its own *Guidelines for Students working with the Six Nations of the Grand River,*^12^ noting an intention to build closer research relationships with Six Nations Polytechnic, the Woodlands Cultural Centre, the Six Nations Language Commission, and Onkwawenna Kentyohkwa. These guidelines noted a “fundamental” need for student researchers to follow the ethics policies of the Six Nations Ethics Committee. In turn, the Manitoulin Anishinaabek Research Review Committee (MARRC) uses the previously mentioned GEAR for research conducted on Manitoulin Island, as well as a customized research ethics application (updated in late 2021)^13^ and a fee-for-service ethics review process (Noojmowin Teg Health Centre of Manitoulin Island, 2003). Maar et al. (2012) put together an *Ethics and Research Review Workbook* to accompany the GEAR and to provide the MARCC and local First Nation communities with a tool to assist in their assessment of research proposals.

Both the First Nations Centre (2007) OCAP® document as well as the Tri-Council (2022) TCPS-2 states that usual ethical requirements for research, such as individual informed consent and confidentiality, still apply to work with Indigenous Peoples (see the TCPS-2 articles 9.9 and 9.16). However, Indigenous Peoples may experience ethical precautions differently. Martin-Hill and Soucy (2005) observed that in their work with First Nations Elders “confidentiality and the use of pseudonyms to conceal the identity of informants were seen as dehumanizing, colonial and patronizing” (p. 8). Bartlett et al. (2007) emphasize the importance of giving credit for Indigenous knowledge to Indigenous People. This may entail attaching identifying data, including full names, to their quotes, a practice that challenges conventional research expectations around confidentiality.

The Tri-Council (2022) notes that “the fit between institutional policies and community customs and codes of research practice may be unclear, requiring researchers to adapt conventional practice or negotiate a resolution” (TCPS-2, article 9.9). OCAP® (FNIGC, 2017) states that any policy divergence must be resolved before research begins, and the TCPS-2 suggests that communication between the institutional REB and responsible community agencies may help in doing so. At times, resubmission to both (or multiple) review bodies may be required.

Where conflicts exist in gaining approval from formal community leaders and customary authorities, the TCPS-2 suggests researchers inform their institutional REB (and presumably allow that REB to suggest a course of action). The TCPS-2 authors (Tri-Council, 2022) suggest it would be inappropriate for an institutional REB to insist on “uniformity between community practices and institutional policies,” or to “impose language and processes that may be experienced as culturally inappropriate or awkward” (article 9.9). For example, when recruiting participants, if it is not culturally appropriate to have individuals sign consent forms, researchers must work with the communities involved and their REB to designate and document culturally relevant processes of informed consent.

The TCPS-2 (Tri-Council, 2022) states that when an REB is regularly asked to review research on topics related to Indigenous Peoples or affecting Indigenous communities in Canada, membership of that REB should be modified to reflect relevant expertise and knowledge, for example, by asking Indigenous or First Nations, Inuit, and Métis scholars or community members to be a part of the review board (Tri-Council, 2022). When less frequent reviews are required, the TCPS-2 authors recommend “consultation with *ad hoc* advisors or delegation to a specialized or multi-institutional REB” as appropriate (article 9.9).

The TCPS-2 authors also suggest researchers be able to provide their REBs with documents that outline attempts at community engagement, if they are not seeking an allowable exception to engagement with the community (see article 9.10), with examples provided. Researchers must clarify with the REB who would be responsible for signing off on research agreements (see articles 9.11 and 9.18, Tri-Council, 2022).

### 3.3 Utility of research

Any research conducted in an Indigenous context should be culturally relevant (Ermine et al., 2004; Inuit Tapiriit Kanatami and Nunavut Research Institute, 2006), and support “cultural preservation and development” (First Nations Centre, 2005, p. 27). This principle is also supported by the Tri-Council (2022) in its TCPS-2 reinterpretation of “Concern for Welfare.” The First Nations Centre (2005) OCAP® document states that local and traditional knowledge should be incorporated into the development of research projects, and notes that “research must respect the privacy, protocols, dignity, and individual and collective rights of Aboriginal Peoples. It must also derive from Aboriginal culture and validation methods” (p. 13).

Indigenous knowledge is embedded in a web of relationships between people (e.g., researchers and participants), but also with animals and plants, with the spirit world, and with the earth itself (Wilson, 2001; Steinhauer, 2002; Ball and Janyst, 2008). Indigenous research principles recognize that cultural concepts, values, and social mores are foundational to Indigenous knowledge and are essential for grounding research (Steinhauer, 2002; Martin, 2003).

### 3.4 Collective ownership

#### 3.4.1 Research agenda

In discussing the research agenda, reference must be made again to Smith’s (1999) book, *Decolonizing Methodologies*, in which she reviews the development of Indigenous research initiatives and ways of articulating an “Indigenous research agenda” at broad and local levels.

In Canadian documents that discuss ethics for conducting research with or alongside Indigenous Peoples, there has been a significant shift in discussions of the research agenda. In the First Nations context, as discussed by the First Nations Centre (2005), research agendas should no longer be shaped by areas of personal, academic, or societal interests, but be inspired by First Nations’ priorities. These concerns are also expressed in Métis and Inuit research ethics dialogues (Inuit Tapiriit Kanatami and Nunavut Research Institute, 2006; Métis Centre of National Aboriginal Health Organization, 2011; ITK, 2018). According to Inuit Tapiriit Kanatami and Nunavut Research Institute (2006), “Communities often complain that there are no tangible benefits for communities who are nearby, or even involved in, the project” (p. 4). Indigenous individuals and communities in Canada have priorities regarding what kinds of projects might serve their needs. In 2020, the Government of Canada released a 3-year strategic plan for *Setting New Directions to Support Indigenous Research and Research Training in Canada: 2019–2022*, guided by four principles: decolonization of research, accountability, equitable access, and self-determination (or, “fostering the right for First Nations, Inuit and Métis Peoples to set their own research priorities,” p. 8).

#### 3.4.2 Research benefits

In general, “the most elegant study design in the world is only as valuable as the impact that it makes in people’s lives” (First Nations Centre, 2005, p. 22). Research conducted with Indigenous Peoples must be explicitly and directly useful or beneficial to participants, with tangible and practical outcomes for them and their communities (Ermine et al., 2004; Kovach, 2021). Community interests should be respected, benefits should be clear, and potential harms should be minimized or eliminated (First Nations Centre, 2005; The First Nations Information Governance Centre, 2014; ITK, 2018). The need for clear and explicit benefits from research is echoed across documents authored by First Nations, Inuit, and Métis groups (First Nations Centre, 2005, 2007; Inuit Tapiriit Kanatami and Nunavut Research Institute, 2006; Métis Centre of National Aboriginal Health Organization, 2011; ITK, 2018). The TCPS-2’s “Mutual Benefits in Research” (Tri-Council, 2022, article 9.13) details the importance of community benefits, which may include education and training, efforts to increase community empowerment, the reclamation of Indigenous identities and cultural property, financial compensation for participation, and the provision of local employment (e.g., via “train-the-trainer” models in clinical or health services research, research assistantships, co-investigatorships) (Indigenous Peoples’ Council on Biocolonialism, 2000; First Nations Centre, 2005; Inuit Tapiriit Kanatami and Nunavut Research Institute, 2006). Researchers should understand from the onset that cultivating collaborative research relationships is time consuming and resource intensive, and funding proposals should reflect development and participation costs (see Inuit Tapiriit Kanatami and Nunavut Research Institute, 2006; Tri-Council, 2022, article 9.11). A barrier to this work is the scarcity of funding sources for collaborative relationship building; by the time the grant is written, it is often too late for a collaborative relationship to be built (i.e., one where community members participate in the design of the study and choosing of research questions). This should be a part of discussions moving forward.

#### 3.4.3 Capacity building

Research should be used for meaningful capacity building (Noojmowin Teg Health Centre of Manitoulin Island, 2003; First Nations Centre, 2005; Inuit Tapiriit Kanatami and Nunavut Research Institute, 2006; Métis Centre of National Aboriginal Health Organization, 2011; The First Nations Information Governance Centre, 2014; ITK, 2018). The Tri-Council (2022) TCPS-2’s article 9.14 addresses this and frames researchers as responsible to incorporate capacity building into their projects, for example, by providing trainings (see the Indigenous Wellness Research Institute National Centre of Excellence, which offers culturally-adapted, online, ethical research trainings)^14^ or helping community members to enhance their skills in research methods, ethical review and monitoring, or intervention delivery. The First Nations Health Authority (FNHA) includes on their website^15^ a variety of “guides, toolkits and workbooks created by First Nations organizations and researchers, aimed at helping communities do research for their own benefit.”

Often, researchers can hire individuals in the community as research assistants, translators, clinicians, or project managers, among other roles. Grant funding may allow research teams to budget for training for students or post-doctoral fellows in the community. The ITK’s National Inuit Strategy on Research (2018) notes that capacity building “also includes investments in built infrastructure and human resources” (p. 27), including working towards an Inuit Nunangat university. In 2018, Canada’s Social Sciences and Humanities Research Council (SSHRC) launched a funding opportunity for multi-disciplinary Indigenous Research Capacity and Reconciliation Grants on National Indigenous Peoples Day, and along with the NSSRC and CIHR, awarded 116 Connection Grants to fund community gatherings, workshops, and events that mobilized and exchanged knowledge on Indigenous research and reconciliation (Government of Canada, 2020).

Capacity building could also involve training research team members in the history and culture of the Peoples with whom they are working, to increase their proficiency in the local language, and to develop skills in Indigenous methodologies (see Lambert, 2015’s Spider Conceptual Framework^16^). Resources for such work include the Intercontinental American Indigenous Research Association, which trains researchers, the public, and Indigenous communities to conduct respectful and ethically sound investigations. As well, the University of New South Wales (UNSW) Sydney has published a web-based searchable database of “anti-colonial” research that is free to peruse, with links to videos and downloadable documents.^17^ The Canadian Institute for Health Information (CIHI, 2020) discusses its own efforts to build fundamental capacity for collaborations with Indigenous Peoples. CIHI notes its intentions to (1) become “culturally responsive” by “training and processes to promote cultural safety and humility,” (2) connect with local, regional and national partners; (3) to “align policies, practices and procedures with Indigenous data sovereignty principles” and (4) to “enable actionable analyses and capacity-building” through collaborative work and increasing the relevance of their “analyses, products, services, training, data infrastructure and tools” for Indigenous partners (p. 5).

#### 3.4.4 Insider and peer researchers

Most Canadian documents aim to guide research conducted by settler researchers employed at settler institutions, and few offer directions to “insider researchers” (in this case, Indigenous scholars who conduct studies with their own or another Indigenous nation). For example, an Indigenous scholar who was themselves apprehended during the Sixties Scoop, may choose to research the impact of the experience on Indigenous adoptees in Canada. Edwards defines someone as a “deep insider” if they have belonged to the community under study for at least five years (Edwards, 2002, p. 71), and Sinclair (2007) defines a “peer researcher” as someone with lived experience of the issue under study. While the label “insider researcher” is often applied to people who have extensive training as researchers, the term “peer researcher” is usually applied to those without previous training, who learn research skills during the study itself. Both types of researchers bring what Kayrooz and Trevitt (2005) describe as “an intimate knowledge of [a community’s] culture, structures, systems and processes” (p. 335).

#### 3.4.5 Collective ownership of information and research

A United Nations resolution (1993/44 of 26 August 1993) acknowledges Indigenous Peoples as holding collective rights. “Indigenous Peoples’ ownership and custody of their heritage,” notes the Sub-Commission on Prevention of Discrimination and Protection of Minorities, “must continue to be collective, permanent, and inalienable, as prescribed by the customs, rules, and practices of each People” (p. 4; cited by Ermine et al., 2004). The Indigenous Peoples’ Council on Biocolonialism (2000) echoes this sentiment in their *Indigenous Research Protection Act* under sections 1.3 (which recognizes the Tribe as exclusive owner of traditional knowledge) and 6.2 m (which affirms the rights of Tribes to hold raw data and research materials and to make decisions about its storage and preservation). Ideally, research done with Indigenous Peoples should heighten their control of information and research processes. The people from whom data are collected should have access to their data, not merely to reports summarizing their data (First Nations Centre, 2005, 2007; Inuit Tapiriit Kanatami and Nunavut Research Institute, 2006; Métis Centre of National Aboriginal Health Organization, 2011; The First Nations Information Governance Centre, 2014; ITK, 2018), with protections for confidentiality and privacy of individual participants (e.g., de-identified datasets, summaries, figures, tables). Inuit Tapiriit Kanatami and Nunavut Research Institute (2006) guide notes that often, “information is placed in a database in a southern institution and communities find themselves unable to gain access, or having to pay for data that they provided” (p. 4).

The First Nations Regional Health Study (First Nations Information Governance Committee, 2007) created a collective ownership protocol for First Nations, and stated that permission must be obtained from local authorities before community- or regional-level data or statistics may be released. In 2010, the Tripartite Data Quality and Sharing Agreement was signed by the First Nations Health Society, now the First Nations Health Authority (FNHA), the BC Ministry of Health, and Health Canada to “continually improve the quality and availability of First Nations Data,” “facilitate the sharing of FNCF^18^ Data in response to research questions approved in accordance with this Agreement,” and to ensure that federally and provincially [BC] held information on First Nations is appropriately “compiled, used and shared” (see Tripartite First Nations Health Plan, 2013; updated in 2022, see Tripartite First Nations Health Plan, 2022). The GEAR document (Noojmowin Teg Health Centre of Manitoulin Island, 2003) affirms “collected data is owned by local communities and agencies” (p. 7).

The TCPS-2 stresses the necessity of determining privacy and confidentiality processes for communities and individuals early in any collaboration (see article 9.16), and, throughout Chapter 9, repeats the importance of consistency among research agreements, informed consent procedures, and disclosure (Tri-Council, 2022). The Indigenous Peoples’ Council on Biocolonialism (2000) *Indigenous Research Protection Act* includes requirements for protecting confidentiality in section 6.2d. CIHI (2020) notes working to align its organizational policies and procedures with principles of Indigenous data sovereignty (e.g., First Nations principles of OCAP; First Nations Centre, 2007), Métis principles of *ownership*, *control*, *access*, and *stewardship* or OCAS (see CIHI, 2020; Indigenous Innovation Initiative, 2021) and *Inuit Qaujimajatuqangit*^19^ or IQ (Tagalik, 2009-2010). The CIHI authors state, “We have learned that these principles reflect “the right of Indigenous Peoples to control data from and about their communities and lands, articulating both individual and collective rights to data access and privacy.” Kukutai and Taylor’s (2016) edited volume on *Indigenous Data Sovereignty* reviews emerging data management practices for how they support Indigenous self-determination, and considers the implications of the UNDRIP for how data are collected, stored, and accessed, and what data handling practices imply “for Indigenous Peoples’ sovereignty over data about them, their territories and ways of life” (p. 2).

#### 3.4.6 Dissemination and publication

Researchers should include opportunities for a community’s leaders or members to review any publications of research involving their community, as well as provide community members with the “right to dissent” by offering divergent interpretations of findings in the publication (First Nations Centre, 2005; Inuit Tapiriit Kanatami and Nunavut Research Institute, 2006; Tri-Council, 2022). Shawn Wilson, author of *Research is Ceremony: Indigenous Research Methods* (2008), suggests “continuous feedback with all the research participants,” supporting each person involved in the study to “check the accuracy of the analysis,” to “elaborate upon ideas,” and “to learn from other participants” (p. 121). The TCPS-2 guidelines note that community representatives in collaborative research should be included when reviewing findings and interpreting data, before final reports or publications are issued (Tri-Council, 2022, see article 9.17).

Any reports, presentations, or publications about community members or knowledge should be provided to that community, regardless of whether they were involved in creating those works or not. Researchers should ensure that community members understand these documents by making translation or plain language versions available (Noojmowin Teg Health Centre of Manitoulin Island, 2003; First Nations Centre, 2005; Inuit Tapiriit Kanatami and Nunavut Research Institute, 2006; Tri-Council, 2022). The Inuit Tapiriit Kanatami and Nunavut Research Institute (2006) guide offers several examples of communications plans for researchers (with benefits and drawbacks to each), such as local radio, focus groups, websites, posters, and written publications. In 2019, MARRC hosted a free research conference^20^ for researchers and community members to discuss research conducted over the previous 5 years. Lunch was provided and the group aimed to share research outcomes and discuss how projects and their findings impacted the community. The IPCB’s *Indigenous Research Protection Act* states that “communications should be carried out in the local language, using translators as necessary” (2000, section 5.1). This assumes financial resources (see “Grant writing,” below), as well as a research review committee with whom researchers are communicating. The latter issue is related to capacity building and should be a part of building collaborative research relationships with communities. Opportunities to discuss authorship and acknowledgment of community leaders should be provided to participating community parties (collective and individual). Similar discussions should occur regarding intellectual property rights and be specified in a research agreement prior to the onset of the research (see TCPS-2, Tri-Council, 2022, article 9.18).

It is suggested that researchers spend time thinking outside of the “box” of peer-review publication when transmitting what they have learned to knowledge seekers. Some Indigenous researchers (e.g., Shawn Wilson, Margaret Kovach) have translated their research through personal narrative, storytelling, and conversation, as well as academic books and articles. Other examples of accessible dissemination methods might include radio communications, websites, posts and reels on social media, videos, and illustrated materials or infographics.

## 4 International application of Canadian lessons

Our goal has been to examine resources supporting the conduct of ethical research with Indigenous Peoples in Canada, with a view to synthesizing the key principles and guidelines, and offering lessons for similarly intentioned international work. With no ambitions towards a systematic global review and synthesis, our scan of relevant peer-reviewed and grey literature identified four broad categories of work relevant to ethical Indigenous research.

The first category is Indigenous scholarly papers and reports. A prime example is the aforementioned seminal book by Smith (1999), but important works also stem from the *United States* (Lomawaima, 2000); *Peru* (Milmaniene, 2009); *Colombia* (Urrego-Mendoza et al., 2017); *Canada* (e.g., Wilson, 2008; Tuck and Guishard, 2013; Drawson et al., 2017; Kovach, 2021); and *Pacific Asia* (Mataira, 2019). These works share a critique of dominant research methodologies and stress the importance of research that originates from within Indigenous knowledge systems, rather than merely incorporating Indigenous perspectives or knowledge into otherwise colonial research. Another theme that emerges is the need for Indigenous sovereignty to extend to research, including control over research data collected from Indigenous Peoples (e.g., Rainie et al., 2019; Walter and Suina, 2019; Walter et al., 2021). The work of the International Work Group for Indigenous Affairs (IWGIA)^21^ on data governance is particularly noteworthy for centering Indigenous Peoples as decision makers regarding how their data are collected, accessed, stored, and used. The IWGIA recommends Indigenous Peoples establish and use policies for community data governance and negotiate mechanisms to ensure that the treatment of any externally stewarded data reflects their Indigenous values (Carroll et al., 2021; Robyn et al., 2022).

The second category is works authored by Indigenous scholars or organizations, some developed with community members and Elders. This category includes works in the *Canadian* context (e.g., Inuit Tapiriit Kanatami and Nunavut Research Institute, 2006; Métis Centre of National Aboriginal Health Organization, 2011); and examples from *South America* (e.g., Meza Guzmán et al., 2021) and from Tribal councils in the *United States* (see Lomawaima, 2000). The work of Huria et al. (2019) represents an important collaboration and synthesis in *New Zealand* and *Australia*. More recently, an international team led by Yuira Celidwen contributed an Indigenous-specific framework of ethical research principles to guide Western-led psychedelic science (Celidwen et al., 2023). These works focus on research approaches that emerge within *specific* Indigenous communities and offer recommendations to incorporate culturally specific values (including territorial knowledge) into research.

The third category is work that proposes formal ethical guidelines or requirements. These works, which closely approximate policy documents, include *Canada’s* TCPS-2 (Tri-Council, 2022) and the Canadian Institutes of Health Research (Canadian Institutes of Health Research, 2010). The former’s span of policy control is bounded by the three Canadian research funding bodies (CIHR, NSERC, and SSHRC) and is mandatory for Indigenous research funded through the Tri-Council. Its closest parallel internationally is Australia’s Australian Institute of Aboriginal and Torres Strait Islander Studies *AIATSIS Code of Ethics for Aboriginal and Torres Strait Islander Research* (updated in 2020), developed in consultation with the National Health and Medical Research Council (NHMRC), the Australian Research Council (ARC) and the National Indigenous Australians Agency (NIAA). Compliance to this Code is required for all research funded by the Australian Research Council (ARC), AIATSIS, or the National Health and Medical Research Council (NMHRC). For the *United States,* applicants seeking funding from the National Institutes of Health (NIH) are encouraged to follow guidance offered by Walters et al. (2019), although this does not appear to be formally mandated or monitored. The United States also released a *Final NIH Policy for Data Management and Sharing*^22^ in early 2023, but official recommendations appear to be in the draft stage:

“The NIH Tribal Consultation Report – NIH Draft Policy for Data Management and Sharing^23^ provides more detail on the Tribal Consultation process relative to the development of the final DMS Policy and NIH’s response. Briefly, three themes emerged from Tribal Nations’ input: (1) Strengthen engagement built on trust between researchers and Tribal Nations; (2) Train researchers to responsibly and respectfully manage and share American Indian and Alaska Native (AI/AN) data; and (3) Ensure research practices are aligned with the laws, policies, and preferences of AI/AN community partners.”

Works in this category (formal ethical guidelines) offer high-level recommendations for research with multiple Indigenous communities, rather than focusing on specific communities in greater depth. Chapter 9 of the TCPS-2, for example, applies to work with First Nations, Métis, and Inuit Peoples (Tri-Council, 2022). For this reason, the implementation of guidelines may require an evaluation of which aspects are relevant locally. Where formal guidelines differ from local or territorial practices, those which are specific to the Indigenous community involved should override practices designed for a broader context (e.g., national or international). Both the TCPS-2 and the *AIATSIS Code of Ethics for Aboriginal and Torres Strait Islander Research* acknowledge that recommendations may sometimes be superseded by local practices as part of the process of tailoring research to meet the needs of the communities involved (see AIATSIS Code of Ethics *for Aboriginal and Torres Strait Islander Research* Section 1.7c and TCPS Section B and articles 9.2 and 9.3).

The last category of work relevant at the international level offers guidelines, policy, and legislation to protect Indigenous rights in research. Such works address environmental protection, health, and social justice. This includes the UNDRIP (2007, p. 28, Article 43) and its policy and legislative adoption in signatory countries. For a national example, see Hepburn’s (2020) review of Peruvian legislation protecting individuals and communities from infringement of rights for purposes of commercialization. Through its Environmental and Social Framework, the World Bank also provided guidance and key principles for funding applicants to its program “ESS7: Indigenous Peoples/Sub-Saharan African Historically Underserved Traditional Local Communities” (World Bank, 2018), although scholars note the program leaves many gaps to be addressed (Lewis and Söderbergh, 2019).

Consistency across internationally relevant documents has developed partly as a result of the widely-read work of Indigenous scholars (e.g., Smith, 1999; Wilson, 2008; Kovach, 2021). Important similarities across International documents include a focus on (a) acknowledging and respecting Indigenous ways of knowing and the use of culturally appropriate research methods and tools; (b) ensuring that research benefits Indigenous communities and addresses their needs and priorities; (c) ensuring that free, prior, and informed consent is obtained (sometimes balanced by collective forms of consent); and (d) empowering Indigenous communities in the research process.

When ethical considerations specific to Indigenous research are compiled into an organizing framework, they are typically articulated as a whole rather than as principles in isolation from each other. This approach is consistent with a holistic worldview. Flexibility is consistently identified as critically important for applying principles and guidelines in different jurisdictional and community contexts. Chapter 9 of Canada’s TCPS-2 highlights such flexibility by acknowledging “the role of community in shaping the conduct of research that affects First Nations, Inuit, and Métis communities” (see section on Context, Tri-Council, 2022). Australia’s previously mentioned *AIATSIS Code of Ethics for Aboriginal and Torres Strait Islander Research* (Australian Institute of Aboriginal and Torres Strait Islander Studies (AIATSIS), 2020) is required for funded research, and encouraged to be “mandatory” for institutions and organizations. The National Health and Medical Research Council (2018b) provides a companion document for the AIATSIS Code and its related Guidelines (National Health and Medical Research Council, 2018a) to support application. Arguments for increasing community participation in Indigenous research in Australia also stress a need for flexibility at the community level in the evolution of the research agenda and processes (Dudgeon et al., 2010; Butler et al., 2022). The Métis Centre of National Aboriginal Health Organization (2011) describes research principles as “not intended to be enforceable rules that must be followed but rather are a well thought out starting point to engage Métis communities in ethical research” (p. 1). Such flexibility also is articulated well in the context of urban/rural considerations, such as in the report from Manitoba’s 2018 Urban Indigenous Health Research Gathering (UIHRG), in which authors reject “a one-size-fits-all approach” for one where researchers “walk alongside communities with one simple instruction: nothing about us, without us” (Morton, 2019, p. 4). In short, the principles and guidelines are framed as adaptable and evolving.

Lastly, while our global scan finds consistency in content and intention, there is considerable inconsistency in terminology, which presents challenges for international comparisons through a policy lens. For example, the terms “principles” and “guidelines” reflect something different from “policy” or “legislation,” with policy being what one “must do” and principles and guidelines reflecting what one “should do.” Policies are formalized requirements that apply to a specific area or task and comprise a written document that establishes a standard by which an institution manages its affairs (University of Wisconsin-Madison, 2022). A policy mandates, specifies, or prohibits conduct to enhance an institution’s mission, ensure coordinated compliance with applicable laws and regulations, promote operational efficiency, and/or reduce institutional risk. Going further, a policy framework typically includes not only policy statements but also the rationale, principles, and guidelines that explain the policy as well as considerations for implementation and evaluation, including procedures to be followed and relationship to strategic directions. While creation of policy entails commitment for evaluation and, ideally, quality improvement activities, evaluation of adherence to policy is often lacking in the international landscape of ethical Indigenous research [see Australian Institute of Aboriginal and Torres Strait Islander Studies (AIATSIS) and The Lowitja Institute (2013), for a notable exception from Australia].

The vast majority of documents related to ethical research with Indigenous Peoples articulate guidelines, recommendations, and/or key principles that establish what “should be.” It may be tempting for jurisdictions and organizations to go beyond guidelines or principles to produce policy with a view to enforcement. However, there are challenges in recommending enforceable policies for formalizing and operationalizing ethical research guidelines with Indigenous Peoples. For example, there are often multiple communities, organizations, and institutions to involve when developing and enforcing a policy or policy framework. The more organizations and institutions involved, the greyer becomes the span of policy control and enforcement. In addition, the need for flexibility and adaptation, given widely varying cultural norms and mores within and across Indigenous communities, including community members living outside their home communities, challenges the use of formal mandatory policies. This diversity is multiplied in an international context.

## 5 A planning guide

We suggest Indigenous scholars, organizations and community leaders/Elders themselves consider the pros and cons of moving from guidelines and principles to formal mandatory policy with respect to Indigenous ethical research. Rather than advocate for Canadian or international policy development *per se*, we draw on our narrative synthesis and recommend a common set of questions that a jurisdiction, organization and/or research team can use when engaging in Indigenous research collaborations (see Supplementary Table S1). To facilitate alignment with international guidelines, we have organized these questions according to the eight research domains identified by Huria et al. (2019) for reporting research involving Indigenous Peoples.

## 6 Conclusion

Academic researchers, regardless of institutional affiliation or context, have a responsibility to conduct ethical research with an intersectional lens that benefits the populations under study. By responding to the aforementioned calls to action, researchers will advance toward fully ethical, respectful, and collaborative research with Indigenous Peoples. It is hoped that this paper will open dialogue at Canadian institutions and beyond regarding how researchers can embed respect for Indigenous cultural protocols and philosophies into research design.

Furthermore, in approaching the shift of research paradigm alluded to in this paper’s introduction, it is important to focus on the strengths of Indigenous communities, and ways to increase Indigenous wellbeing, rather than produce statistics about negative issues or problems faced. Much of the scientific literature has produced disparity-focused research rather than strengths-based research, which reinforces the subordination of Indigenous Peoples by bolstering stereotypes rooted in white supremacy. There is an inherent power difference in the “researcher–researched” dynamic (First Nations Centre, 2005) that must be minimized so Indigenous nations may lead and direct research that affirms their sovereignty and supports their cultural survival.

### Positionality statements

**Dominique Morisano, PhD, CPsych** (she/her) is of Italian/Balto-Slavic descent and a dual Canadian/US citizen raised in rural Connecticut. She is a clinical psychologist and Adjunct Professor at the University of Toronto/University of Ottawa and engages in teaching, practice, research, and consultation in North America and Europe. Her research has focused on research ethics, addiction and mental health services, goals/motivation, implementation science, and more recently plant medicines. As a child, she became very interested in learning about and protesting injustices against Indigenous Peoples in North America, and asked her parents to bring her to local powwows to connect with area Indigenous Peoples and learn about the histories and traditions of those who had traditionally populated the lands she occupied. She continued her learning exploration through and beyond graduate school, studying the intersections of spirituality and science, including Native American religions; volunteering at Montreal’s First Nations Friendship Centre; participating in ceremonies and forming healing relationships with several Elders (in both the North and South); and via ongoing collaborations with Indigenous colleagues. While working as an independent scientist at the Centre for Addiction and Mental Health (CAMH), she was seconded to an appointment with the unit formerly known as Aboriginal Engagement and Outreach (now Shkaabe Makwa), and continued collaborating with her colleagues there after leaving.

**Margaret Robinson, PhD** (she/her) is a Mi’kmaw (L’nu) scholar and a member of Lennox Island First Nation. Her mother’s ancestors were Scottish and Irish, and Margaret holds status under section 6.2 of the Indian Act of Canada. Raised in Sheet Harbour, in the Eskikewa’kik district of Mi’kma’ki, Margaret completed her undergraduate studies at Saint Mary’s University in Halifax/Kjipuktuk, and earned a PhD in Theology from the University of Toronto in the homeland of the Huron-Wendat, the Seneca, and the Mississaugas of the Credit. She now works as an Associate Professor at Dalhousie University in Mi’kma’ki, where she holds the Tier II Canada Research Chair in Reconciliation, Gender, and Identity. Margaret identifies as two-spirit, bisexual, and queer, and her research with sexual and gender minority people examines substance use, mental health, and how culture and identity support wellbeing. Margaret served on the Indigenous Advisory Board of the Institute of Indigenous People’s Health at the Canadian Institutes of Health Research, and now serves on the Tri-Council’s Reference Group for the Appropriate Review of Indigenous Research.

**Brian Rush, PhD** (he/him) is of Irish and Welsh descent and born in Canada in 1951, He is a health services researcher whose work has focused on the planning and evaluation of mental and substance use health services and systems with a focus on the synthesis and translation of evidence into practice. This work has involved significant consultations with Indigenous communities and organizations working on their behalf and specific recommendations for improving access and coordination, inclusive of traditional land-based healing (e.g., provincial strategic plan for the province of Manitoba). Other work has aimed to develop and evaluate tools and processes for culturally relevant and trauma informed screening and assessment tools for Indigenous Peoples seeking mental health and substance use health services and support [e.g., Centre for Addiction and Mental Health (CAMH) Aboriginal Engagement and Outreach (now Shkaabe Makwa]. Recent work has also been in support of Indigenous communities (from the North to the South) to gain access to traditional entheogenic substances for healing and overall individual and community wellness. Through all of these and other experiences Brian has gained a deep appreciation of Indigenous ways of knowing and how to work collaboratively from a position of respect and reciprocity.

**Renee Linklater, PhD** (she/her), is of Anishinaabe and Scottish/English ancestry and a member of Rainy River First Nations in Northwestern Ontario. She has much lived experience as a First Nations person in Canada. Two generations of her family attended Indian Residential Schools—both her grandparents and her mom - and as a baby, she was apprehended by the Children’s Aid Society and became part of what is now known as the 60s scoop. Renee reconnected with her family and community in 1988. Over the last 35 years she has developed very strong relationships in her personal, community, and professional life. In her academic studies, she explored the impacts of trauma and delved deep into the cultural knowledge that exists within Indigenous communities. Renee has extensive experience with Elders, healers, and in ceremonies. She has a thorough understanding of Indigenous research ethics and protocols. At the Centre for Addiction and Mental Health (CAMH), she is Senior Director of Shkaabe Makwa and leads the first hospital-based Centre in Canada designed to drive culturally-responsive systems initiatives to achieve health justice and wellness for First Nations, Inuit, and Métis through the advancement of research, workforce development, and innovative healing models that harmonize traditional knowledge and medical expertise. She has over 25 years of experience working with Indigenous healing agencies and First Nation communities. Renee has worked across the health and education sectors as a frontline worker, program evaluator, curriculum developer, educator/trainer, and researcher. She is an international speaker on trauma and healing and is the author of *Decolonizing Trauma Work: Indigenous Stories and Strategies and editor of Connected in Creation: A Collection of Lived experience through Cultural Expression*.

## Author contributions

DM, MR, and RL contributed to conception, design, and outline of the research project and manuscript. BR contributed to the manuscript. DM wrote the first draft of the manuscript. DM, MR, and BR wrote sections of the manuscript. All authors contributed to the collection of source materials, contributed to manuscript revision, read, and approved the submitted version.

## Funding

MR was supported by the Tier 2 Canada Research Chair in Reconciliation, Gender, and Identity (award no. 950-232722).

## Footnotes

^1^Consistent with the literature to be summarized we use the terms “principles” and “guidelines” interchangeably, both being distinct from policies which are mandatory in nature.

^2^See https://camh.ca

^3^Biomedical research is beyond its scope, although such research is also associated with serious problems (e.g., using leftover blood samples from Indigenous Peoples without consent; First Nations Centre, 2005)

^4^The words of the Indigenous Peoples’ Health Research Centre (IPHRC; Ermine et al., 2004) are relevant here: “Not all knowledge and viewpoints have been recorded, particularly as they are embedded in the oral tradition of the Indigenous community” (p. 11). Many documents, practices, and pieces of oral knowledge exist off the Internet.

^5^Two of the authors identified key threads or principles within the sources (MR; DM), and shared these with the team. The team then discussed the principles and categorized them into broader themes (MR; DM; BR). The results were checked by a fourth team member (RL). An Indigenous author was involved in each of these steps.

^6^The authors wish to note that Indigenous Peoples have objected to the use of terminology such as “First Nations, Inuit and Métis Peoples of Canada” as undermining the sovereignty of Indigenous nations by framing them as possessions of Canada.

^7^See also the *AIATSIS Code of Ethics for Aboriginal and Torres Strait Islander Research* (Australian Institute of Aboriginal and Torres Strait Islander Studies or Australian Institute of Aboriginal and Torres Strait Islander Studies (AIATSIS), 2020).

^8^A note that Inuit elders and knowledge keepers traditionally do not accept tobacco as an offering as it is not used ceremonially; see https://carleton.ca/indigenous/wp-content/uploads/Guidelines-for-Working-with-Indigenous-Elders.pdf

^9^ https://tarp.indigenousto.ca

^10^See the National Association of Friendship Centres at nafc.*ca.*

^11^OCAP® is a registered trademark of the First Nations Information Governance Centre (see FNIGC, 2017; https://fnigc.ca/ocap-training/)

^12^ https://linguistics.humanities.mcmaster.ca/wp-content/uploads/sites/13/2020/12/Guidelines-for-students-working-with-the-Six-Nations-of-the-Grand-River-final.pdf

^13^ https://www.noojmowin-teg.ca/images/2021_10_17_-_MARRC_Ethics_Application_Form_BLANK.pdf

^14^See http://iwri.org/research-area/research-training/

^15^See https://www.fnha.ca/what-we-do/research-knowledge-exchange-and-evaluation/research-resources

^16^See https://www.americanindigenousresearchassociation.org/mission/spider-conceptual-framework/

^17^See https://www.anticolonialresearchlibrary.org/library/

^18^FNCF – First Nations Client File. The First Nations Client File is a cohort of BC Resident First Nations People registered under the Indian Act, and their unregistered descendants for whom entitlement-to-register can be determined.

^19^“*Inuit Qaujimajatuqangit* (IQ is the term used to describe Inuit epistemology or the Indigenous knowledge of the Inuit). The term translates directly as that which Inuit have always known to be true’” (Tagalik, 2009-2010, p. 1).

^20^ https://www.manitoulin.com/conference-seeks-to-develop-stronger-communication-on-indigenous-research/

^21^The IWGIA is part of the International Indigenous Data Sovereignty Interest Group, which developed the CARE Principles for Indigenous Data Governance in consultation with Indigenous Peoples, scholars, non-profit organizations, and governments. The CARE Principles are as follows: Collective Benefit, Authority to Control, Responsibility, and Ethics. These principles complement the approach of the FAIR Guiding Principles for scientific data management and stewardship, which proposes that data be Findable, Accessible, Interoperable, and Reusable. (Wilkinson et al., 2016).

^22^Available at: https://grants.nih.gov/grants/guide/notice-files/NOT-OD-21-013.html (Accessed October 13, 2023).

^23^Available at: https://osp.od.nih.gov/wp-content/uploads/Tribal_Report_Final_508.pdf (Accessed October 13, 2023).
