# Supplementary material for: Conducting research with Indigenous Peoples in Canada: ethical and policy considerations
Source: Front Psychol. 2024 Jan 10;14:1214121. doi: 10.3389/fpsyg.2023.1214121 (PMC10866143; doi:10.3389/fpsyg.2023.1214121)
Supplement: Supplementary file 1 [file Table_1.pdf]

Supplementary Material

Conducting Research with Indigenous Peoples in Canada: Ethical and Policy Considerations

Dominique Morisano, PhD, CPsych*, Margaret Robinson, PhD, Brian Rush, PhD, Renee Linklater, PhD

*** Correspondence:**
Dr. Dominique Morisano, CPsych
dominique.morisano@utoronto.ca

Supplementary Table (or online Appendix)

Supplementary Table 1. **Questions to Guide Planning and Implementation of Indigenous Research.**

**Governance**
How will your project embody respect for a range of Indigenous authorities, such as Elders, hereditary and elected leaders, and those working in communities?
How will you share power within the project beyond striking a community advisory board?
Which Indigenous agencies or authorities should be engaged during the consent process? What form of approval or oversight will they have across the life of the project?
How are Indigenous partners involved in identifying potential harms and developing a plan to minimize or eliminate those harms?
What reviews or permissions will be needed before work begins? Which Indigenous ethics boards are available to review the proposal?
What Indigenous community guidelines will direct and inform the project?
Which templates have been selected as most appropriate for guiding research agreements?
**Prioritization**
How will the centrality of Indigenous priorities be demonstrated across the life of the project?
How will you identify community research priorities?
Has the research timeline been designed to accommodate Indigenous communities' decision-making processes and customs, for example, related to seasons of the year, holidays, community events, or communal or horizontal decision-making methods?
How will you incorporate and prioritize Indigenous worldviews and epistemologies?
How will your project balance individual and collective issues (e.g., rights, data access)?
Which health equity impact assessment tool will you select to identify and address potential health impacts of your research?

**Relationships**
How will you engage Indigenous communities prior to determining the research questions, designing the study, applying for funding or implementing the work?
Have you developed an existing partnership with community or organization members before developing research proposals?
What decision-making process will you use when conflicts arise? How will this plan prioritize Indigenous decision-making?
What roles will Indigenous people have on the research team?
How might the research impact the connections between humans, other animals, and the environments that sustain them?
How will diverse interests and perspectives within Indigenous communities be reflected in the project (e.g., youth and Elders, multiple genders, sexual identities, multiple understandings of group belonging)?
How will mutual respect be incorporated and communicated throughout the project?
What other researchers are engaged in research with the Indigenous communities with which you plan to partner? How might their work support or impact the work you hope to do?
**Methodologies**
What certifications or review processes are available from regional Indigenous research agencies?
How will you facilitate communication between institutional review boards and community review boards to reduce delays, ensure research plans are culturally appropriate, and increase collaboration capacity?
How will you build in time to allow for a culturally appropriate pace for the work?
How will you ensure the research is culturally relevant and supportive?
How will the project incorporate local and traditional knowledge, culture, values, and validation methods?
What measures can you use to ensure consistency between research agreements, informed consent procedures, and data disclosure plans?
What methods will be used that support collaborative research, including techniques developed by Indigenous researchers or designed to center community participation?
**Participation**
How might the project engage groups or individuals who are frequently denied research opportunities due to their vulnerability or marginalization?
Who needs to be involved in co-developing privacy and confidentiality processes with communities and individuals?
How will you incorporate elements of oral culture into the group and community consent process?
How will your project engage and support the use of Indigenous language?

How can consent be engaged in secondary data analyses, especially of data from Indigenous communities?
How will you inform prospective participants about the community collaboration and data sharing agreements in your ongoing consent process?
How will the project's approach to participant confidentiality acknowledge the individual Indigenous source of knowledge or teachings?
**Capacity**
Have you budgeted for and secured resources for all stages of the project, including:<br>• gaining a solid familiarity with the local history, customs, and processes involved in conducting the research?<br>• compensating for Indigenous-focused ethics and other reviews, including legal if required?<br>• staff time to develop an agreement?<br>• supports to co-create a research agreement and study designs and support ongoing collaborative implementation?<br>• hiring community members and supporting Indigenous students?<br>• building language skills on the research team? and<br>• communications in the local language?
Which formal and informal research protocols and guidelines will you need to know thoroughly to conduct the work in a culturally appropriate and approved way?
What tangible benefits and practical outcomes will the participants and their communities obtain?
How will you support increased capacity to engage with research?<br>Benefits may include education and training, efforts to increase community empowerment, the reclamation of Indigenous identities and cultural property, financial compensation, and the provision of local employment (e.g., via "train-the-trainer" models in clinical or health services research, research assistantships, co-investigatorships).
What options are available for hiring and purchasing equipment locally?
What peer support is available from scholars conducting similar work at your institution or in your area of expertise?
How will you support your own institution to involve Indigenous scholars or community members in their review board as ad-hoc or regular members?
How will you support the control of information and research processes by Indigenous Peoples within and beyond your project?
How will the research team advance their skills in forming community partnerships, conducting culturally safe research, and adopting anti-oppressive practices?
**Analysis and interpretation**
Have you developed a data quality and sharing agreement with relevant community representatives? How will you include both individual and collective perspectives?
Which community authorities will have access to identifiable personal data?
How will you engage the community to interpret the findings?

What processes will enable community members to offer divergent interpretations on the data, and to include dissenting opinions in publications?
What measures will enable academic and community researchers to check the accuracy of your data analysis and ensure it is correctly contextualized?
**Dissemination**
How will your project support the deposit of raw materials or data, including de-identified data, working papers or similar data products in a secure repository, accessible to Indigenous participants and their communities?
How will you recognize and affirm the community's ownership over their traditional knowledge?
How will you engage communities broadly when sharing findings?
How will your knowledge translation or exchange activities maximize access to findings while also protecting sensitive information?
How will you facilitate the review of manuscripts and other data sharing products by the community before submission for publication or public release?
How will final reports, presentations, and publications drawing upon community members' knowledge be provided to communities?
What resources need to be budgeted for Indigenous language translation, infographics, plain language products, and accessible materials to be able to share data findings with all community members?
What agreement have you made regarding the sharing of scholarly credit (e.g., authorship or acknowledgement) and intellectual property rights?
What role can personal narrative, storytelling, conversation, radio communications, websites, social media, videos, and general audience products have in the knowledge translation and dissemination plan?
